# Supplementary material for: Asymmetric Synthesis of Quaternary Hydantoins via a Palladium-Catalyzed Aza-Heck Cyclization
Source: J Am Chem Soc. 2025 Nov 14;147(49):44692–8. doi: 10.1021/jacs.5c16022 (PMC12703750; doi:10.1021/jacs.5c16022)
Supplement: Supplementary file 2 [file ja5c16022_si_002.zip › All NMR FID Files/S30/S30_AllNMR/HRMS/DWatson_Idowu_TDI01050.pdf]

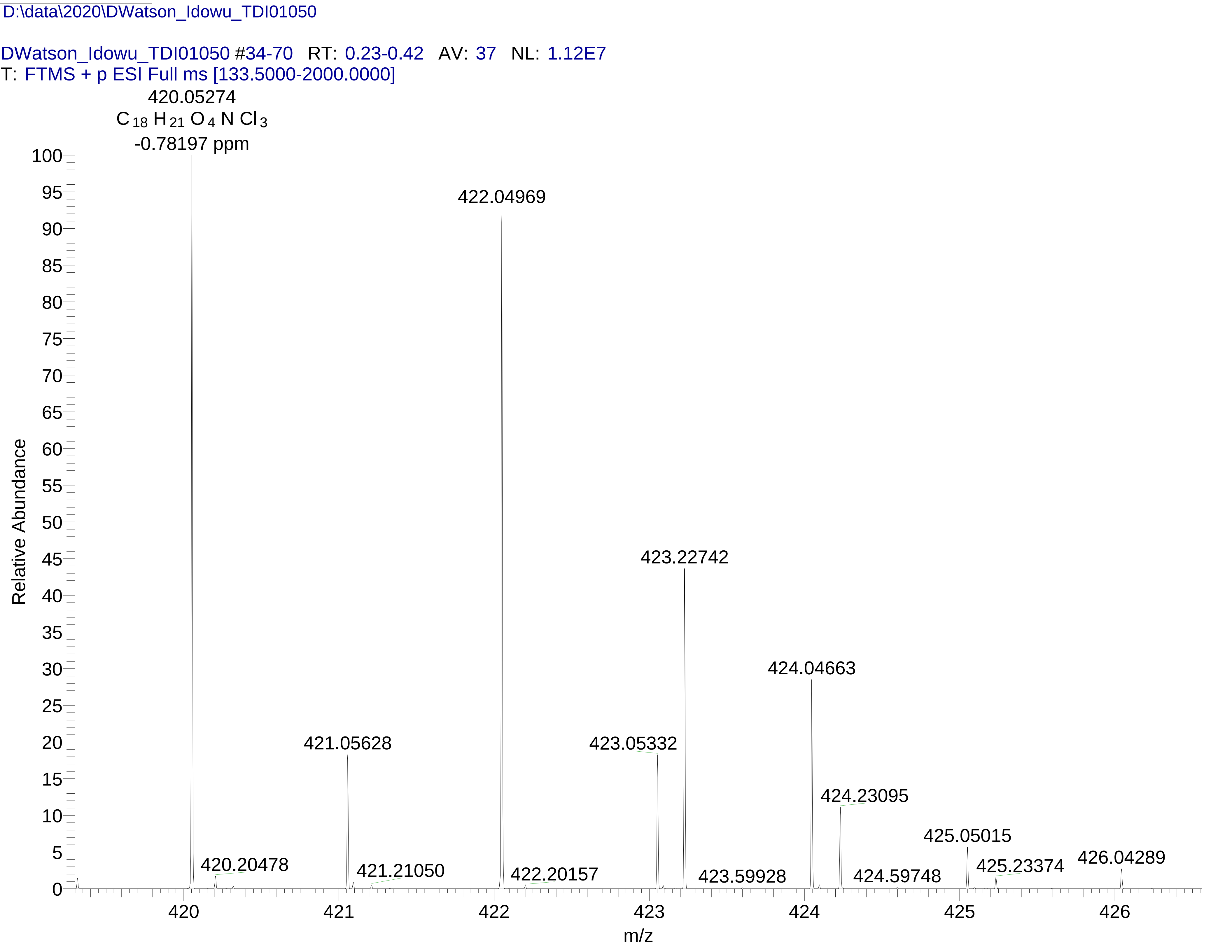
D:\data\2020\DWatson_Idowu_TDI01050
DWatson_Idowu_TDI01050 #34-70 RT: 0.23-0.42 AV: 37 NL: 1.12E7
T: FTMS + p ESI Full ms [133.5000-2000.0000]
420.05274
$C_{18}H_{21}O_4NCl_3$
-0.78197 ppm
422.04969
423.22742
424.04663
421.05628
423.05332
424.23095
425.05015
420.20478
421.21050
422.20157
423.59928
424.59748
425.23374
426.04289
Relative Abundance
m/z
